# Supplementary figures and images for: Influence of analysis conditions for antimicrobial susceptibility test data on susceptibility rates
Source: PLoS One. 2020 Jun 23;15(6):e0235059. doi: 10.1371/journal.pone.0235059 (PMC7310835; doi:10.1371/journal.pone.0235059)

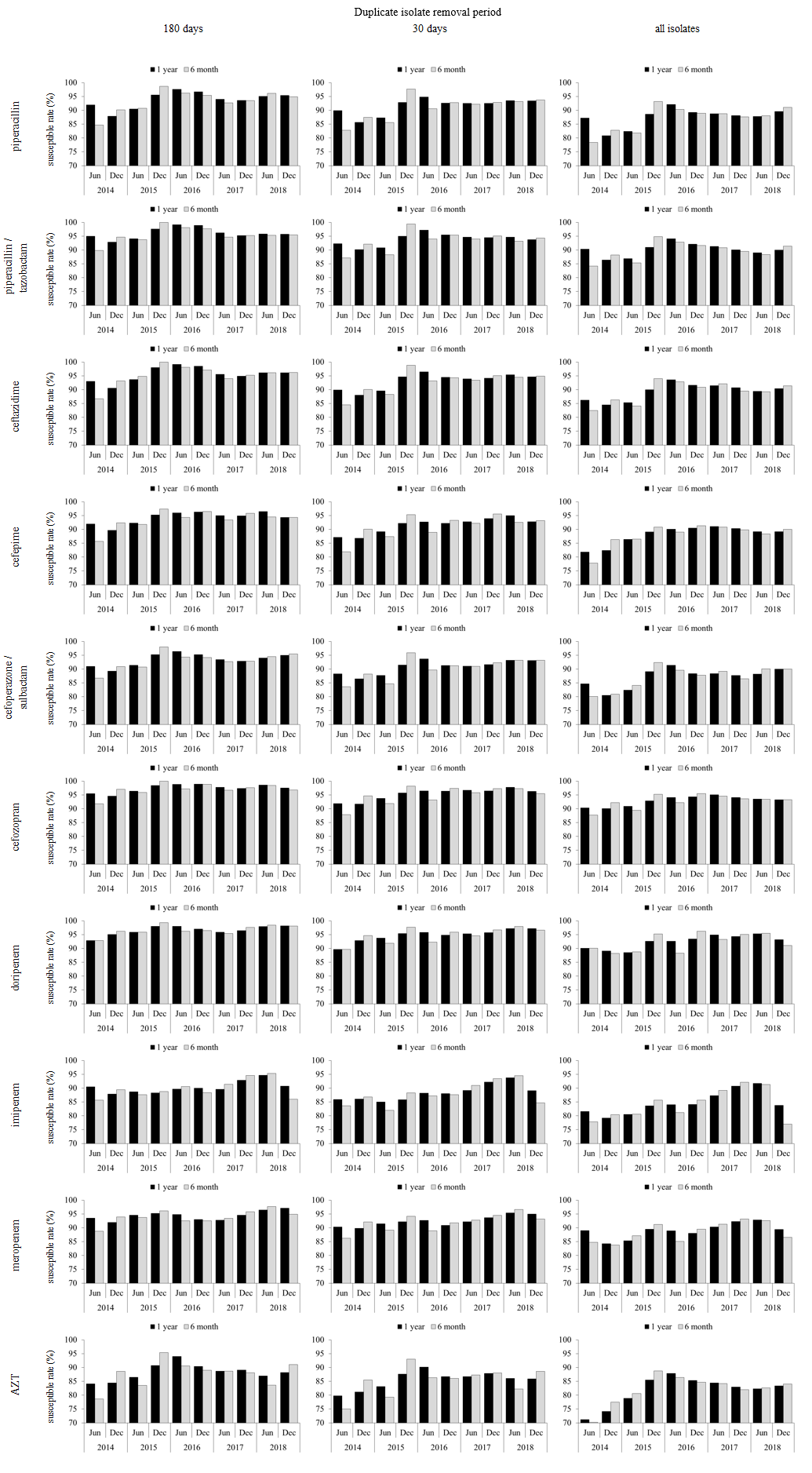

Supplement: S1 Fig — (TIF) [file pone.0235059.s001.tif]
