# Supplementary material for: Influence of analysis conditions for antimicrobial susceptibility test data on susceptibility rates
Source: PLoS One. 2020 Jun 23;15(6):e0235059. doi: 10.1371/journal.pone.0235059 (PMC7310835; doi:10.1371/journal.pone.0235059)
Supplement: Attachment — Submitted filename: Response to Reviewers.docx [file pone.0235059.s003.docx]

Reviewer 1:

Thank you for your helpful suggestions. I appreciate your time and effort spending for our manuscript. I have all of your suggestions into my revision.

1. Pseudomonas aeruginosa are notorious for their ability in the upregulation of intrinsic resistance mechanisms, especially in the presence of antibiotic use. Inclusion of another organism, e.g. Enterobacteriaceae, may provide a more complete picture of how varying parameters affect susceptibility rates in antibiogram.

→ We added *Escherichia coli* and *Klebsiella pneumoniae* to target bacteria. And we recreated data and rewrite manuscript. Although the final conclusions have not changed significantly, it has become clear that the magnitude of influence of parameters differs according to the bacterial species.

2. Also, only beta-lactam antibiotics were included in this study, thus it will be interesting to see if similar changes of susceptibility rate are observed for the other classes of antibiotics, e.g. fluoroquinolones, aminoglycosides.

→ We added 20 more antibiotics to target antibiotics, including 2 fluoroquinolones, 3 aminoglycosides, and others. And we recreated data for 29 target antibiotics in 9 groups, and rewrite manuscript.

Minor issues:

1. Table 1 does not add much information. The total number of isolates can be put into table 2 in an additional column.

→ As a new Table 1 was inserted to the manuscript, Table 1 and Table 2 were renamed to Table 2 and Table 3, respectively. Table 2 and Table3 had become complicated because of the increase in number of target bacterial species and target antibiotics. Therefore, we did not integrate Table 2 and Table 3 into one table.

2. Dripenem should be spelt doripenem.

→ I replaced “dripenem” with “doripenem”.

3. p. 13 Line 222 cefzoplan should be spelt cefozopran

→ I replaced “cefzopran” with “cefozopran”.

In “Revised Manuscript with Track Changes”, deleted parts are shown in strikethrough and red text, and added parts are shown in blue text. Please understand that corrections to minor grammatical or calculation errors in the manuscript that do not affect the conclusion are not shown in red or blue text.
