# Supplementary material for: Influence of analysis conditions for antimicrobial susceptibility test data on susceptibility rates
Source: PLoS One. 2020 Jun 23;15(6):e0235059. doi: 10.1371/journal.pone.0235059 (PMC7310835; doi:10.1371/journal.pone.0235059)
Supplement: Attachment — Submitted filename: Response to Reviewers.docx [file pone.0235059.s004.docx]

Thank you for your helpful suggestions. I appreciate the time and effort you spent reviewing our manuscript. I have taken all of your suggestions into account in my revision.

Reviewer 1:

1. The statistical significance of the susceptibility rate difference of the various ‘duplicate isolate removal periods’ should be calculated instead of the absolute change in %.

→ We used the chi-square test to assess for associations between susceptibility rate and the duplicate isolate removal periods. In addition, differences in sensibility rates between the long and short data collection periods were also tested using the chi-square test. (Line 129)

2. The ranking order of antibiotic susceptibility rate has little clinical or interpretation significance and can be omitted entirely to simplify table 3.

→ We agree with your assessment. We abandoned the idea of ranking order and have removed Table 3.

3. Subgroup analysis could include comparisons between Pseudomonas aeruginosa and the Enterobacteriaceae, as well as between various antibiotic groups. These further analyses could reveal that Enterobacteriaceae susceptibility data are less affected by the change in period of deduplications for beta-lactam antibiotics, and aminoglycoside group antibiotics are not significantly affected by the deduplication periods etc.

→ We showed that the duplicate isolate removal periods significantly affected the susceptibility rate only in *Pseudomonas aeruginosa* β-lactams. We also compared differences in susceptibility rates across data collection periods using coefficients of variation to highlight differences between β-lactams and other antimicrobials in *P. aeruginosa*, and between *P. aeruginosa* and two other bacterial species.

Reviewer 2:

1. It is not clear what impact the “rank order of antimicrobial susceptibility” has to a clinician.

→ We agree with your assessment. We abandoned the idea of ranking order of antimicrobial susceptibility and have removed Table 3.

2. Table 1: CTX is given as the abbreviation for two cephalosporins, this should be corrected before publication. Throughout the entire manuscript (and namely in tables and figures), again, DORIPENEM and CEFOZOPRAN need spelling corrected. This is noted in Table 1 with doripenem, FIGURE 1 for both, and also in Table title text.

→ I apologize for my carelessness. I have corrected Table 1 and other relevant sections in the manuscript.

3. Line 390: This reviewer would avoid using terms such as “a few percent” as it is not customary reporting..

→ I corrected the phrase "at a few percent" to "under 3%" in the relevant sections. (Line 295)

4. Discussion: Somewhere in the discussion the authors should add some reference to the fact that antibiograms are used for empiric selection of antimicrobials pending full culture results, therefore, the clinician should fully grasp that the antibiogram susceptibility results for any pathogen and antibiotic may vary, even tremendously, based on the interval of time (duration) for which duplicate isolates are removed from the calculation/percentages.

→ We have inserted the appropriate text and the reference you pointed to in the “Effect of duplicate isolate removal periods” section of the “Discussion.” (Line 331)

5. Line 461: Would remove term “settle down” and chose alternate wording.

→ I have corrected the phrase "the emergence of new susceptibility profiles, which would eventually settle down" to "eventually leading to a new susceptibility profile" in the relevant section. (Line 357)

6. Line 499: Last word, “required.” would replace with “recommended.”.

→ I have corrected the phrase "required" to "recommended" in the relevant section. (Line 390)

In “Revised Manuscript with Track Changes,” deleted parts are shown in strikethrough and red text. Text that has been added is shown in blue text. Please consider that minor grammatical corrections to the manuscript that do not affect the conclusion are not shown in red or blue text.
