# Supplementary material for: Influence of analysis conditions for antimicrobial susceptibility test data on susceptibility rates
Source: PLoS One. 2020 Jun 23;15(6):e0235059. doi: 10.1371/journal.pone.0235059 (PMC7310835; doi:10.1371/journal.pone.0235059)
Supplement: Attachment — Submitted filename: Response to Reviewers.docx [file pone.0235059.s005.docx]

Dr. Herman Tse

Academic Editor

PLOS ONE

June 5, 2020

PONE-D-19-32239R2

Influence of analysis conditions for antimicrobial susceptibility test data on susceptibility rates

PLOS ONE

Dear Dr. Herman Tse,

Thank you for your helpful suggestions. I appreciate the time and effort you spent reviewing our manuscript. We have taken all of your suggestions into account in my revision. I hope that our edits and the responses we provide below satisfactorily address all the issues and concerns you have noted.

1. L86-87: Gram stain is named after its inventor, and thus "Gram" should always be spelt with a capital "G".

→ In accordance with your advice, we have changed “gram-negative” to “Gram-negative”. (Line 83, 84)

2. L85-86: It is not necessary to introduce the abbreviated forms of bacterial names as this is the convention in biomedical literature.

→	We thank you for this comment. Accordingly, we have deleted the abbreviated forms. (Line 85, 86)

3. L92 & table 1: In biomedical literature, colistin should be classified as a polymyxin or a nonribosomal peptide.

→ We appreciate your comment on this point. Accordingly, we have changed colistin classification to “polymyxins”. (Line 92, Table 1)

4. L116: Consider changing to "As it is possible that isolates from the same patient among long-term in-patients may undergo ..."

→ Thank you for your suggestion. We have changed the sentence you pointed out as your suggestion. (Line 115)

5. L119: "... to prevent results from being affected ..."

→ Thank you for your advice. We have changed the sentence by adding “from”, which I forgot to add. (Line 118)

6. L147: space missing from E. coli

→ Thank you for your advice. The error you pointed out has been corrected. (Line 146)

In “Revised Manuscript with Track Changes,” deleted parts are shown in strikethrough and red text. Text that has been added is shown in blue text.

Again, thank you for giving us the opportunity to strengthen our manuscript with your valuable comments. We have worked to incorporate your feedback and hope that these revisions persuade you to accept our submission.

Sincerely,

Yasutoshi HATSUDA

Faculty of Pharmacy, Osaka Ohtani University

Tondabayashi-shi, Osaka, Japan

hatuday@osaka-ohtani.ac.jp
